# Experimental and Numerical Investigation of the Flexural Behavior of Reinforced-Concrete Beams Utilizing Waste Andesite Dust

**DOI:** 10.3390/ma17174413

**Published:** 2024-09-07

**Authors:** Fuat Korkut, Memduh Karalar, Ali Motameni, Essam Althaqafi, Nebi Özdöner, Yasin Onuralp Özkılıç

**Affiliations:** 1 Department of Civil Engineering, Van Yüzüncü Yıl University, Van 65090, Turkey; 2 Department of Civil Engineering, Zonguldak Bulent Ecevit University, Zonguldak 67100, Turkey; 3 Department of Metallurgical and Materials Engineering, Middle East Technical University, Ankara 06800, Turkey; 4 Civil Engineering Department, College of Engineering, King Khalid University, Abha 61421, Saudi Arabia; 5 Department of Civil Engineering, Necmettin Erbakan University, Konya 42090, Turkey; 6 Department of Technical Sciences, Western Caspian University, Baku 1001, Azerbaijan

**Keywords:** bending performance, finite-element analysis, rupture attitude, reinforced-concrete beam, andesite powder

## Abstract

During the process of cutting andesite stones, the waste mud is kept in powder form once fully dried. It is difficult to store the waste that is produced as a consequence of the extensive utilization area and consumption of andesite. Thus, eliminating waste storage challenges and incorporating these wastes into the economy are crucial. For this reason, this study examined the effects of waste andesite dust (WAD) on the flexural behavior of reinforced-concrete beams (RCBs) using experimental testing and 3D finite-element modeling (FEM) via ANSYS. Thus, different rates of WAD up to 40% were used to investigate the influence of the WAD rate on the fracture and bending behavior of RCBs. While the RCB with 10% WAD had a slightly lower load-bearing and ductility capacities, ductility capacities significantly drop after 10% WAD. At 40% WAD, both the load-bearing capacity and ductility significantly reduced. Based on the experimental findings, using 10% WAD as a replacement for cement is a reasonable choice to obtain eco-friendly concrete. Moreover, the outcomes of 3D FEM were also compared with those of experiments conducted using ANSYS v19 software. The displacement values between the test and FEM findings are quite similar.

## 1. Introduction

The disposal of rubbish without submitting it for recycling has become a major global environmental issue. Still in its early stages, however, is the utilization of the waste products that have amassed in developing countries. Reusing waste resources to create sustainable materials has been the subject of several studies [1,2,3,4,5,6,7]. The escalation of waste production is a consequence of many factors, including rapid economic expansion, advancements in technology, industrialization, urbanization, population growth, and improved standards of living in various countries. The challenges arising from the growing volume of garbage require the implementation of a waste management strategy that strives for waste-free or less-wasteful production and consumption, and this has led to using them in concrete or cement-based construction materials.

Andesite is a type of volcanic rock frequently used for building pavements, curbstones, steps and stairs, and window frames. Andesite is present in several hues, including gray, dark gray, black, reddish, brownish, and pink [8]. Upon conducting a literature review on the use of waste andesite dust (WAD) in different material types, it becomes evident that WAD finds application in diverse material categories, including cement [9], conventional concrete [10,11,12,13], and geopolymer composite [14] production. Notably, the investigations conducted in these studies primarily emphasize the examination of mechanical properties. To responsibly dispose of these wastes and return them to the economy, Ceylan and Davraz [15] looked at the feasibility of employing them in cement-based composites. For this reason, an experimental investigation was conducted to examine the mechanical and microstructural features of cement-based composites made by substituting cement with waste andesite at weight percentages of 5%, 10%, 15%, 20%, 25%, and 30%. Upon the conclusion of this research, it was noticed that the incorporation of WAD into the mixes resulted in an increase in the pore structure, which was attributed to the porous nature of the dust. Consequently, substituting cement with WAD at lower replacement rates, specifically up to 15%, resulted in more favorable outcomes. To examine the mechanical and microstructural characteristics of cement-based composites made by substituting cement with waste andesite at weight percentages of 5%, 10%, 15%, 20%, 25%, and 30%, Özkan and Ceylan [16] conducted another study. It was found that the compressive strength values improved with an increasing WAD substitution ratio and declined with sample age. In a separate study, Çelikten [14] examined the mechanical and microstructural characteristics of geopolymer mortars synthesized using waste alkali-activated materials. To examine these characteristics, geopolymer mortars were produced using waste activated slag with varying concentrations of sodium hydroxide (NaOH) as the alkali activator. The findings indicate that longer curing durations are associated with higher values of flexural strength and compressive strength. The activation of waste andesite powder with a 12 M NaOH solution leads to notably high compressive and flexural strength values. Soykan et al. [17] conducted another study. The objective of this study was to examine the usefulness of slate and WAD as concrete aggregates for interior and exterior coatings, as well as for decorating and restoration purposes in building construction. This study investigated the impact of altering the proportions of aggregates at 25%, 50%, 75%, and 100% in two distinct types of cement, namely CEM II/B-M (P-LL) 32.5 N and CEM I 42.5 R. Based on these findings, the use of WAD has demonstrated the potential to enhance the mechanical and physical characteristics of concrete compared to the control concrete. Notably, the incorporation of a WAD resulted in a 12.3% increase in compressive strength, an 8.6% increase in splitting tensile strength, and an 8.6% increase in bending strength. In a study conducted by Eken [18], an examination was undertaken to explore the feasibility of substituting raw fine aggregates with basalt, diabase, and andesite natural stones. The substitution percentages ranged from 10% to 40% in relation to the mass of sand. The impact of incorporating basalt, diabase, and andesite into concrete was assessed by conducting strength and durability tests on the specimens after 7, 28, and 60 days. The samples containing andesite, diabase, and basalt had compressive strengths that were 67%, 63%, and 64% greater than those of the reference sample, respectively. The study conducted by Uzun [19] examined the use of waste produced during the cutting process of andesite stones as aggregate and filler material in asphalt concrete, with a focus on achieving a smooth geometric form. In the beginning, limestone samples were created with a consistent granulometry curve using varying proportions of 3%, 4%, 5%, 6%, 7%, and 8%. Similarly, andesite stone samples were prepared with asphalt cement, including proportions of 5%, 6%, 7%, 8%, and 9%. The experimental specimens were fabricated using fine aggregate andesite, coarse aggregate limestone, fine aggregate limestone, coarse aggregate andesite, and asphalt cement. The aggregate/bitumen ratio used in the study ranged from 4% to 8%. The investigation revealed that the samples including coarse aggregate limestone and fine aggregate andesite exhibited greater stability values. Specifically, this particular combination required a binder content of 5.45%, whereas the other mixture required a binder content of 5.87%. The study conducted by Uzun and Terzi [20] examined the use of andesite obtained from the shaping process of andesite blocks as a mineral filler in asphalt mixes. Among all the mixes examined, the andesite mineral filler with a bitumen percentage of 6% exhibited the greatest degree of stability. In another investigation, Orhan et al. [21] investigated the impact of weathering on the geomechanical properties of andesite in three specific locations in Ankara. This investigation involved the use of various analytical techniques, including optical microscopy, X-ray diffraction analysis, major element analysis, pressurometer tests, physicomechanical tests, and seismic refraction. The geotechnical evaluations indicate that the region does not exhibit any issues related to bearing capacity and consolidation settling. Another investigation was conducted by Ceylan and Davraz [15]. Andesite waste powders were compared with F-class fly ashes as a mineral ingredient for use in concrete. The use of mineral additives, namely andesite waste powder, Seyitömer fly ash, and Tunçbilek fly ash, in cement mixtures was carried out at varying weight percentages of 10%, 15%, and 20%. It was shown that despite the use of additions, the additive samples achieved the desired strengths even if they did not have the compressive strength of the control samples. The C55/67 and C70/85 strength classifications were observed. The optimal proportion of andesite waste powder for concrete manufacturing was identified as 20% in terms of the displacement rate. The other investigation was performed by Özkan [22]. In this investigation, the author examined the effects of integrating a ternary hybrid fiber composition and substituting part of the cement with WAD on the mechanical characteristics of cement-based composite materials. To achieve this objective, WAD was employed in conjunction with ternary hybrid fibers in cement-based mixtures, replacing 0–30% of the cement’s weight at four different levels. The experimental results showed that higher elongation and deflection capabilities resulted from increasing the substitution rate, but decreasing the WAD substitution rate had a favorable impact on strength measures. An experiment by Bayraktar et al. [23] examined how diatomite powder and WAD affect the microstructural, durability, and mechanical properties of environmentally friendly alkali-activated lightweight composites. The combination with 50% waste andesite sand exhibited the lowest strength loss (19.3%) and enhanced the freezing and thawing endurance of sustainable alkali-activated lightweight mixtures. Özkan [22] studied the impact of inserting ternary hybrid fibers and substituting cement with waste andesite dust on the mechanical characteristics of cement-based composites. To achieve this objective, WAD was applied in conjunction with ternary hybrid fibers included into cement-based blends. This was carried out by replacing 0–30% of the weight of cement at four different levels. Testing showed that increasing the WAD replacement rate increased elongation and deflection while decreasing it increased strength. In addition to the above studies, there are various studies in this field in the literature [24,25,26,27].

The above analysis reveals a lack of comprehensive research on the behavior of RCBs constructed with various combinations of WAD, all of which possess the same compressive strength, when subjected to structural stresses. There are also no studies that have been conducted to investigate the impact of utilizing WAD rather than cement has on the impact of the flexural behavior of the beam as a structural element. Furthermore, there is a dearth of research examining the suitability of using WAD in conventional concrete for sustainable construction.

## 2. Study Objectives

This study investigates the impact of different rates of WAD on the bending behavior of RCBs. To achieve this objective, a total of five RCBs (300 × 400 × 2000 mm) were constructed inside the designated testing area. Primarily, RCBs with a 0% WAD beam were manufactured in a thorough manner. Subsequently, various rates of WAD were used in RCBs to assess the influence of the WAD rate on the fracture and bending behavior of RCBs. The literature shows a dramatic decline in strength when a specific percentage is surpassed; hence, ratios up to 40% were chosen [18,22]. Thus, the percentages of WAD utilized to replace the cement were as follows: 0%, 10%, 20%, 30%, and 40%. The objective of this study is to assess the influence of varying rates of WAD on the fracture and bending characteristics of RCBs.

In addition, another objective of this study was to validate the FEM outcomes by test results. To achieve this goal, ANSYS-based [28] FEM representations of RCBs are demonstrated. When it applies to FEM investigations, the FEMs of RCBs have specified bending and fracture levels, and these fracture and bending levels are quite close to the test results.

## 3. Experimental Test Configuration

### Characteristics of the Materials and Examination Method

**Figure 1:**
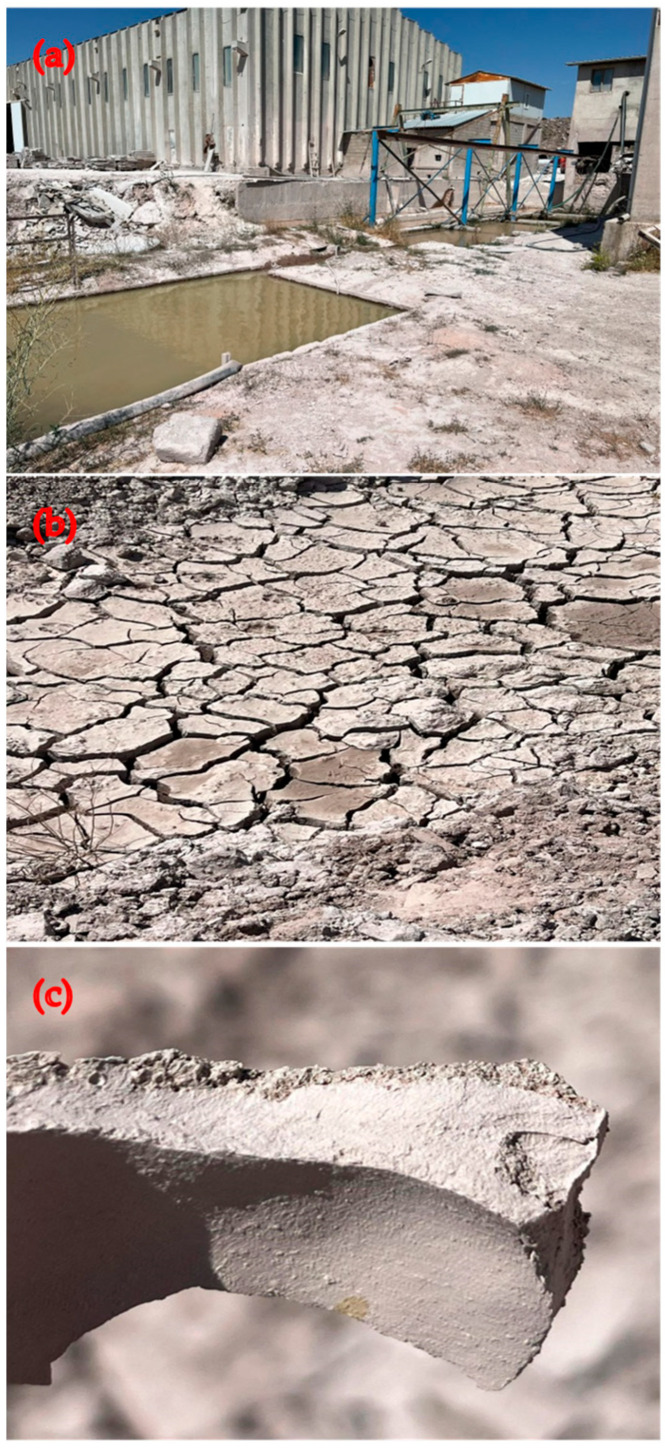
WAD collection area, (**a**) collection pool, (**b**) the state of receding water, (**c**) dried WAD.

The andesite processing facility located in Ankara, Turkey, provided the WAD. The remaining dust is created when andesite stones are chopped and after other procedures. Water is used in the cutting process; therefore, the manufacturing process produces watery leftovers that are collected in waste lakes around the building. Water in the pools evaporates due to exposure to sunlight and atmospheric conditions in the outside environment. Following their accumulation in the lake, the dust residues that sink to the bottom are removed with an operator and placed in a different, open location for storage, as shown in Figure 1. The facility finds it quite challenging to remove much rubbish. Insufficient research has been conducted on the assessment of waste at the location. Because of this factor, the residues are steadily growing, forming peaks. This condition has significant adverse impacts on the ecosystem.

Recycling WAD derived from discarded products is widely recognized as a crucial practice for safeguarding the environment. Moreover, the occurrence of cracks and bending in the RCBs of concrete structures subjected to vertical loads introduces significant risks in terms of assessing their future performance and ensuring their structural integrity. To achieve the objective of this study, different rates of WAD were considered in various RCBs to explore the potential for reusing these waste materials. A total of five series of RCBs incorporating different rates of WAD were arranged at the experimental site. An RCB was developed for each test due to the experimental setup’s size and weight. In light of the fact that every experimental configuration was also evaluated in relation to the finite-element model, it was determined that a single test setup was enough for every experiment. The purpose of this arrangement is to investigate the influence of WAD rates on the fracture and bending behavior of RCBs. Only one beam-bending test was conducted for each RCB series. However, to establish a final assessment of these shortcomings, it was essential to determine the mechanical characteristics of concrete via experimentation conducted at the designated test site. Hence, the distinctive qualities of concretes were determined by the implementation of experimental evaluations, which were discussed in this section. Table 1 provided details on how the concrete samples were set up at the test location.

**Figure 2:**
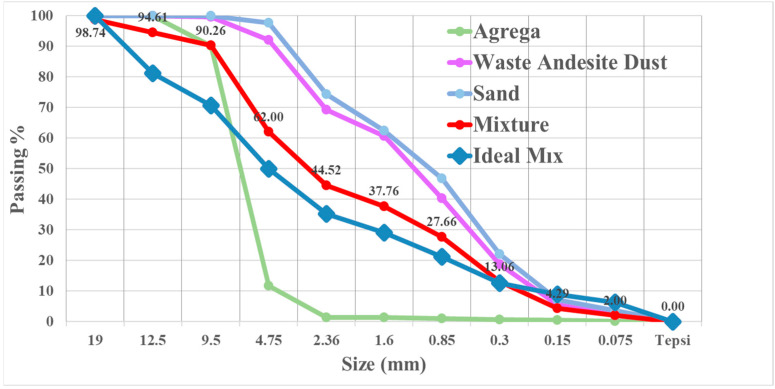
Distribution of aggregates according to subdivision dimensions.

The compositional proportions of the concrete series are shown in Table 2. The absence of WAD may be seen in Table 2 within the context of RCBs. Furthermore, the rates of WAD in the concrete mixture are 10%, 20%, 30%, and 40%. The cement and andesite chemical and physical analyses are given in Table 3. It is possible that the concrete may benefit from extra qualities due to the high calcium concentration of WAD. Additionally present were magnesium, lead, and iron, all of which have the potential to play a role in the concrete’s properties [13]. Due to andesite-sludge particle sizes increasing porosity, cement and admixture-powder particle sizes may affect filler properties [13]. The distribution of aggregate sizes is shown in Figure 2. In addition, this curve agrees with previous research findings in the literature [15].

For the purpose of this investigation, a standard pan mixer is used. When using a rotating concrete-drum mixer, the appropriate quantities of the coarse and fine aggregates, as well as powder components (cement and WAD), were mixed together. They were combined in dry circumstances for a period of two minutes, then more water was added, and the mixture was stirred for another three minutes. For the purpose of achieving the necessary consistency, the concrete mixture was stirred for a final three minutes following the addition of the superplasticizer. The mixing rates that were used for the investigation were 100 revolutions per minute (RPM). After the concrete mixes, including WAD, are placed on the mixer, the machine runs the mixtures for 180 s to organize them. Following each blending operation, the equipment and its corresponding supplies undergo a thorough washing process. In addition, a systematic blending procedure is used to ensure consistency in the production of specific concrete mixtures. Subsequently, slump-flow experiments were conducted for every concrete mixture. This research employed a 20 cm × 10 cm × 30 cm metallic shaft to deposit and compress concrete material to test the consistency of freshly created concrete in a highly participatory manner. Concrete is layered three times on the shaft before being squeezed. This study introduces a metal shaft and describes the procedure for conducting a slump-flow test. During the testing process, sufficient concrete samples are collected from concretes containing WAD.

**Figure 3:**
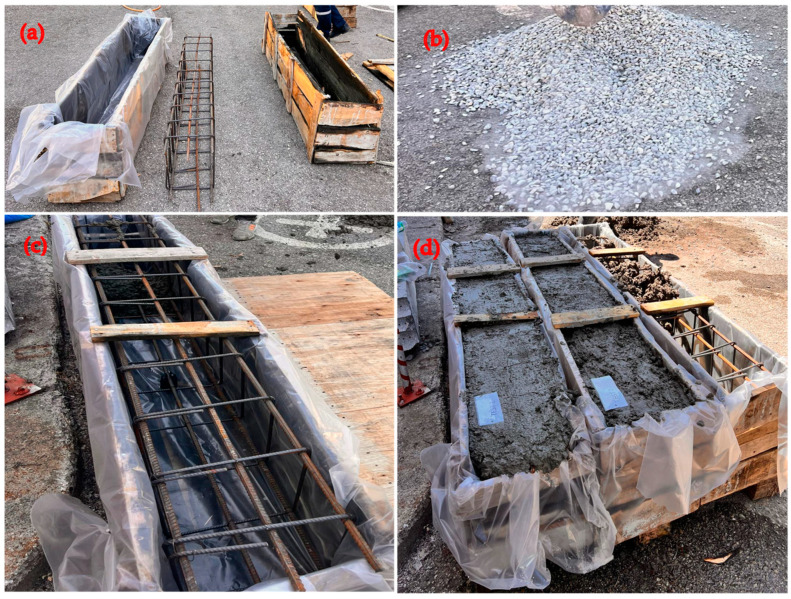
Preparation of RCBs, (**a**) preparation of molds, (**b**) materials, (**c**) reinforcement in the RCB, (**d**) setting up concrete.

It should be noted that as the rate of WAD in the mix increases, there is a corresponding rise in the slump values of the concrete. A slump measurement of 10 cm was observed in the combination containing 0% WAD, and this value increased proportionally with the addition of WAD. The influence of the WAD rate on the consistency of the concrete results in a 13 cm slump-down at a WAD rate of 10%. A 15 cm droop-down is observed when 20% WAD is added to the concrete mixture, and maximum 17 cm and 18 cm slump-downs are noted when 30% and 40% WAD rates are applied. Specifically, when the WAD rate in the concrete mix is increased from 10% to 20%, there is a significant improvement in the concrete consistency. Furthermore, it may be observed that an elevated proportion of andesite dust in the concrete mixture leads to a notable rise in the level of consistency. The preparations of RCBs are given in Figure 3.

To determine the compressive strength of the concrete samples, cube specimens of 150 mm × 150 mm × 150 mm were used. A selection of three distinct concrete samples was taken from a pool of five different concrete samples, resulting in a total of 15 samples being used for the cubes. The water pool was set at 20 °C for 28 days. Many studies have been conducted to investigate the impact of curing temperature on concrete performances, since it affects the entire performance of the material [29,30]. It has been discovered that the compressive strength of concrete samples was significantly affected by the curing temperature and that the compressive strength rises as the curing temperature raises [30]. However, in this study, since the focus was on the effect of WAD on the flexural behavior of the reinforced-concrete beam, the effect of different curing temperatures was not considered in detail.

An average compressive strength of 23.5 MPa was determined with no WAD. Three concrete-cube tests with a 10% WAD rate had an average compressive capacity of 22.2 MPa. Moreover, this study specifically presents the effects of compressive capacity for a WAD rate of 20%. It is evident that an increase in the WAD content in concrete reduces the compressive strength. Concrete with 20% WAD exhibited a compressive strength of 20.9 MPa. Compressive strength was rapidly decreased with 20% WAD. Concrete with 30% WAD exhibited a maximum capacity of 18.1 MPa, while the average compressive strength for concrete with a WAD rate of 40% was determined to be 16.8 MPa. It is evident that an increase in WAD results in a reduction in the compressive capacity value of concrete when examining the relationship between these five different rates of WAD.

## 4. Modeling RCBs Using 3D Nonlinear FEM

According to the literature, FEM analyses are crucial to experimental research, notably to avoid test failures and enhance test equipment. Therefore, it is still necessary to make FEM predictions earlier than the actual tests that are carried out. It is possible for engineers to make use of such FEMs, which are capable of simulating the behavior of the structure, in order to save the enormous costs and considerable amount of time that are necessary for performing experimental tests. When constructing 3D FEMs of RCBs, the forms and boundary requirements of the actual RCBs created at the test site were considered in the 3D FEM. The 3D FEM for RCBs is shown using ANSYS v19 software. In the visualization of RCBs, various FEMs often depict the entire component to accurately represent the actual geometry. To achieve this objective, two reinforcements with a size of 12 mm were considered for the compression part of the RCB, while three reinforcements of the same dimension were considered for the tension section of the RCB. Also, 8 mm reinforcements were used for stirrups. Within the context of FEM, the SOLID65 and LINK80 elements are often used to model concrete structures and simulate compression and tension forces. In our finite-element model, concrete behavior was simulated using a nonlinear material model based on the von Mises yield criterion. Compared to simple linear models, this approach allows for a more accurate representation of concrete’s complex stress–strain relationship. We employed a bilinear approximation of the Mises plasticity model for the compression and tension components to balance computational efficiency with accuracy. Material properties obtained through experimental evaluations, such as the elasticity modulus, shear modulus, Poisson’s ratio, and density, are then assigned to the ANSYS v19 software using specific codes [31,32,33,34]. While meshing the RCBs, the lines were interconnected to form a mesh structure. Convergence issues occurred during analysis. Therefore, the FE mesh was altered numerous times, and a new mesh was constructed to obtain the correct result and avoid program failure. For this 3D model, 50 mm mesh was appropriate. Subsequently, all constituent components were intricately interconnected, culminating in the completion of the three-dimensional volumetric meshing process.

## 5. Structure of Investigational Tests

**Figure 4:**
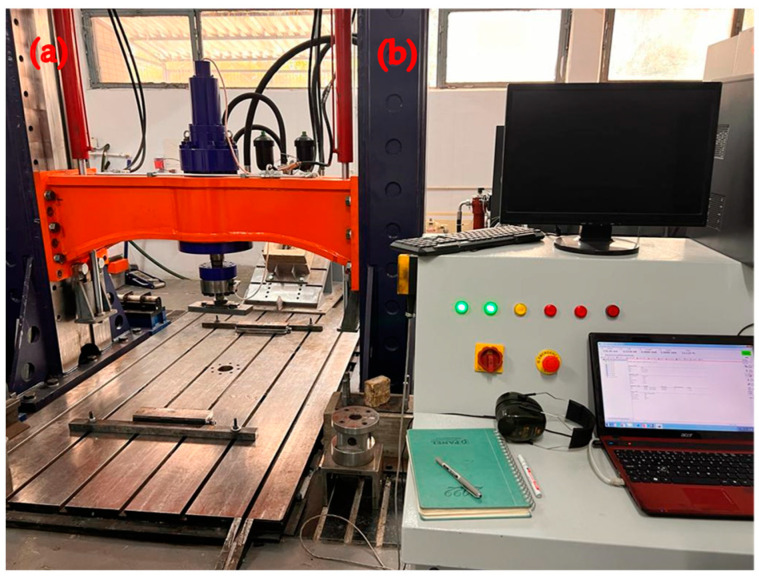
Overview of experimental setup, (**a**) test frame, (**b**) control panel.

An extensive overview of the test framework for RCBs is provided in this section. Figure 4 displays the apparatus used to examine the fracture and bending properties of RCBs. The RCBs are positioned with a pin support on one side and a roller support on the other side. This phenomenon allows the RCBs to undergo deformation when subjected to external loads. To quantify displacement values at the RCBs, a linear variable differential transformer (LVDT) with a displacement capability of ±250 mm was positioned, as shown in Figure 4. Additionally, an actuator was positioned to provide an axial load on the RCBs with a load capacity of 1000 kN. A data logger was used to record the applied vertical load and displacements, and the data were stored on a hard drive connected to the equipment through a computer. The procedure of calibration is an essential step that guarantees the precision and dependability of measurements. A third-party approved calibration laboratory calibrates the equipment or instrument.

## 6. Investigational Test Outcomes and Analysis

This section presents the effect of the WAD rate on the fracture and bending attitude of RCBs. When these RCB fractures are specifically examined, it becomes clear that the bending and fracture characteristics of each WAD rate vary from one another. This study examines the performance of five different RCBs, as shown in Table 1.

### 6.1. Experiment 1: Analysis of the Fracture Behavior and Load–Deformation Characteristics of RCBs Containing 0% WAD

**Figure 5:**
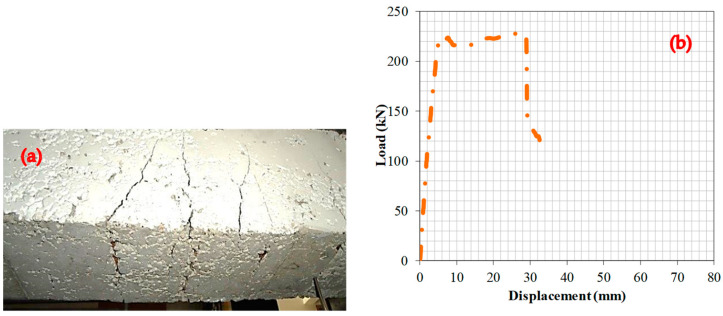
RCBs under perpendicular loads, (**a**) cracking and bending behaviors, (**b**) load–deformation attitudes of RCBs with 0% WAD.

The impact of WAD proportions on the bending performance of RCBs was considered. To achieve the desired objective, the percentage of WAD incorporated into the RCB was set at 0%. There was evidence of cracking occurring toward the lower portion of the RCB. Furthermore, the RCB indicates that the maximum spacing between perpendicular fractures is 150 mm (Figure 5). For RCBs containing no WAD, the load–deformation graphic is shown in Figure 5. It shows the linear continuation of the deformation line up to a load of 178 kN. The load–deformation graph has a consistent trend from 178.0 kN to 216.4 kN, indicating a linear relationship between the applied load and the resulting deformation. However, beyond the load of 216.4 kN, the deformation experiences an upward trajectory. After that, deformation increases linearly until the maximum load (228.5 kN), at which time a 2.75 cm deformation is observed at the maximum perpendicular load. Following the application of a force of 228.5 kN, it can be seen that while the load has decreased, there is a significant improvement in the deformation.

### 6.2. Experiment 2: Analysis of the Fracture Behavior and Load–Deformation Characteristics of RCBs Containing 10% WAD

**Figure 6:**
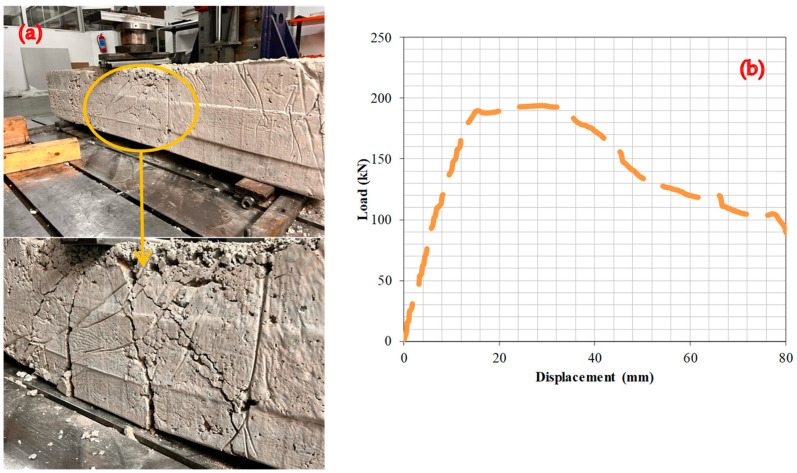
RCBs under perpendicular loads, (**a**) cracking and bending behaviors, (**b**) load–deformation attitudes of RCBs with 10% WAD.

This section focuses on the evaluation of the bending behavior of RCBs with 10% WAD. Figure 6 reveals the presence of significant vertical cracks located near the midpoint of the RCB. Furthermore, significant bending fractures were observed in the lower portion of the RCB. The influence of the WAD rate on the cracking attitude of the RCBs is shown by this result. The deformation line has a linear trend and persists until a load of 189.6 kN is reached. Following the application of a load of 188 kN, the direction of the deformation line undergoes modifications, resulting in a nonlinear trajectory. Between 189.6 and 193.9 kN of load, the deformation line increases linearly. However, despite the increase in the perpendicular load to 193.9 kN, there is a significant increase in the deformation value. It is apparent that the inclusion of WAD in the RCB leads to an improvement in the rate of deformation, resulting in an increase in maximum deformation under a vertical load.

### 6.3. Experiment 3: Analysis of the Fracture Behavior and Load–Deformation Characteristics of RCBs Containing 20% WAD

**Figure 7:**
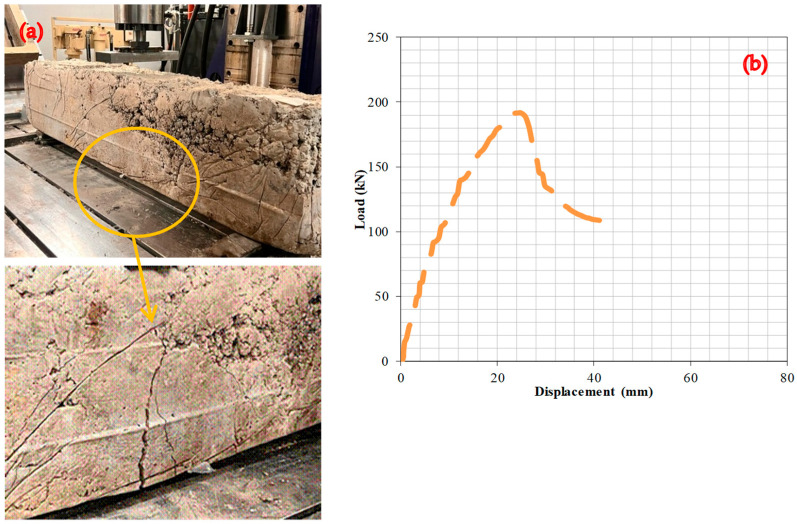
RCBs under perpendicular loads, (**a**) cracking and bending behaviors, (**b**) load–deformation attitudes of RCBs with 20% WAD.

This section focuses on the assessment of the effects of 20% WAD rate on the fracture and deformation behavior of RCBs. Bending fractures in RCBs can be detected in Figure 7, with the greatest number of bends occurring in the middle of the RCBs. Although there is a correlation between the RCB content of concrete made with either 10% or 20% WAD, more-noticeable fracture widths can be observed in concrete produced with 10% WAD. These results clearly demonstrate the effect of the rate of WAD on the cracking behavior of RCBs. The detection of linear deformation stroke occurred throughout the load range of 0–188 kN. Additionally, the middle of the RCB experienced 24.8 mm deflection at a maximum loading of 191.7 kN. Subsequently, a nonlinear deformation line was discovered after a load of 191.7 kN, and the RCB reached its maximum bearing capacity.

### 6.4. Experiment 4: Analysis of the Fracture Behavior and Load–Deformation Characteristics of RCBs Containing 30% WAD

**Figure 8:**
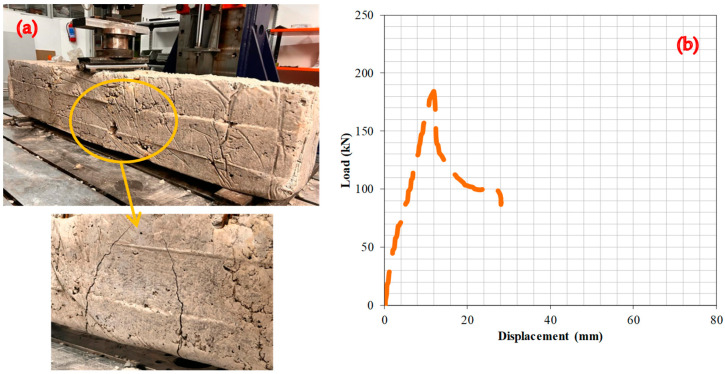
RCBs under perpendicular loads, (**a**) cracking and bending behaviors, (**b**) load–deformation attitudes of RCBs with 30% WAD.

In this section, the effect of a WAD rate of 30% on the bending attitude of the RCBs is given and examined in further detail. Figure 8 shows the detection of significant shear and perpendicular cracks in the RCB. Significant perpendicular fractures are located in the middle of the RCB in Figure 8, and it is crucial to determine the fracture attitude of RCBs containing 30% WAD on the basis of these fractures. The deformation line exhibits a linear relationship throughout the load range from 0 to 184.3 kN. At a load of 184.3 kN, a deformation of 11.9 mm was observed. Upon the application of a load of 184.3 kN, the deformation–load relationship of the RCB transitions from a linear to a nonlinear trajectory.

### 6.5. Experiment 5: Analysis of the Fracture Behavior and Load–Deformation Characteristics of RCBs Containing 40% WAD

**Figure 9:**
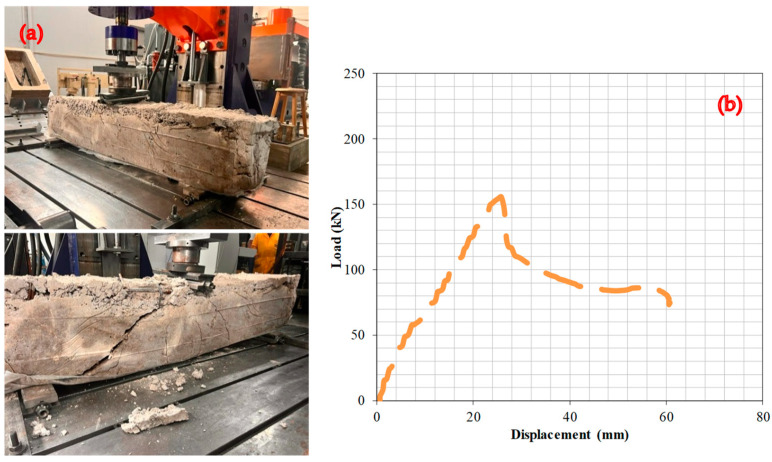
RCBs under perpendicular loads, (**a**) cracking and bending behaviors, (**b**) load–deformation attitudes of RCBs with 40% WAD.

Figure 9 presents the test findings illustrating the effects of RCB with a 40% WAD rate. The deformation line exhibits a linear rise within the load range of 0–155.6 kN and reached the maximum capacity at 25.8 mm; then, the capacity was directly decreased. This specimen showed the least capacity among all specimens.

**Figure 10:**
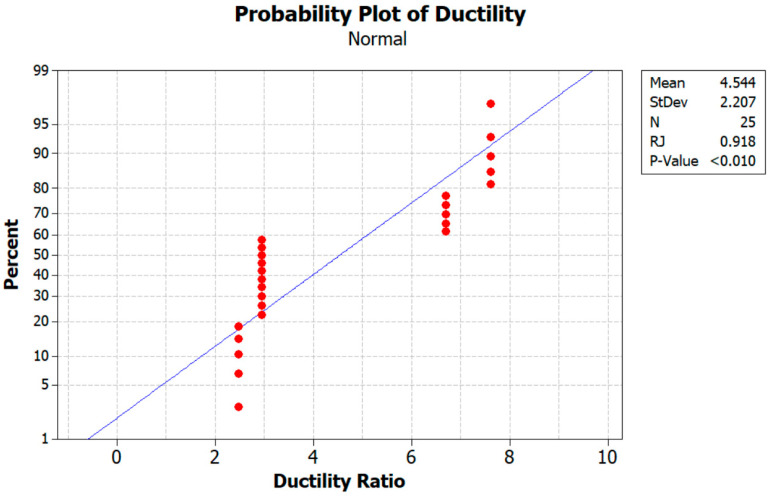
Probability plot of ductility.

Furthermore, according to an analysis of the test findings, the load-carrying capability of the RCB decreases as the ratio of WAD in the mixture increases. Numerous other investigations in the literature are comparable to this one [9,10]. It is mostly because of the diluting of the cement that the efficiency factor drops with the amount of andesite present. This is a phenomenon that can hardly be counteracted by the modest pozzolanic activity of andesite [9]. Existing research demonstrates that the increase in porosity is directly linked to the substitution of WAD, which appears to be the underlying cause of the reduction in strength. In Figure 10, the probability of ductility is given. As observed from Figure 10, the mean value and standard deviation obtained were 4.544 and 2.207. Furthermore, in the relationship between ductility and stiffness, the maximum value of Pmax is found, as indicated by Table 4.

## 7. Fracture Characteristics

**Figure 11:**
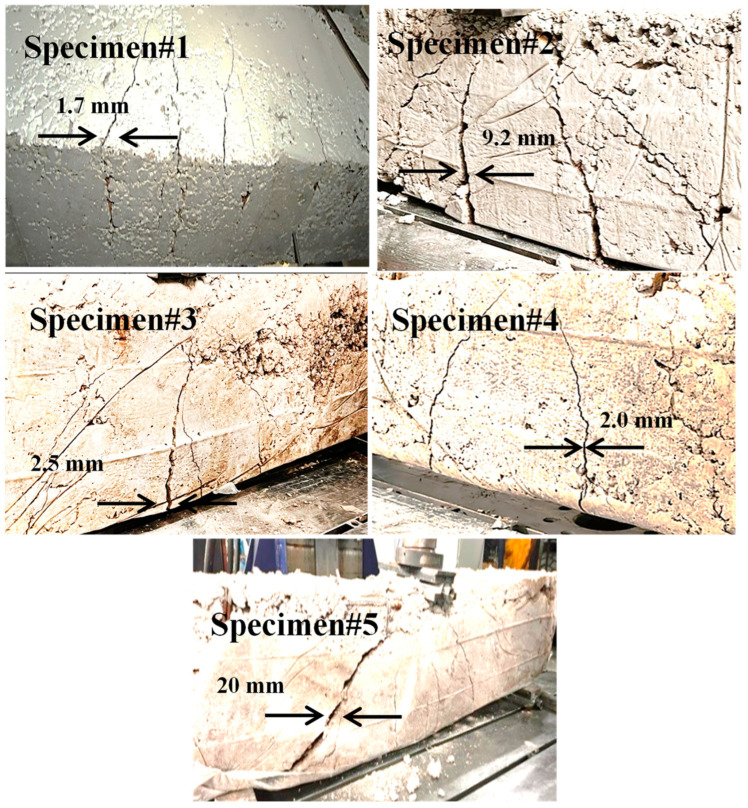
Crack propagation.

**Figure 12:**
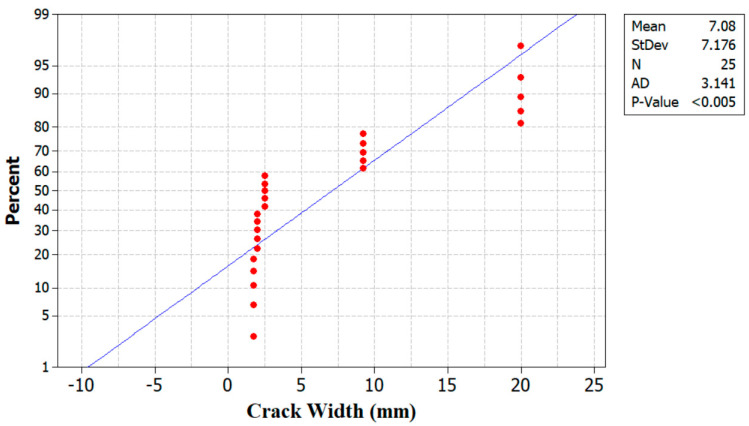
Probability plot of crack width.

The failure mode of the tested beams is displayed in Figure 11. The crack width in the RCBs increased from 1.7 mm to 20 mm on average when the volume of WAD content was increased from 0% to 40%. Conversely, there is a noticeable rise in the quantity of cracks present in the beams, as shown in Figure 11. As the rate at which WAD is substituted into the mixtures increases, voids and weak spots are created locally in the cementitious matrix. As a result of the lack of adhesion at the cement–aggregate interface, these voids and weak spots can lead to the production of new cracks under load and the rapid propagation of pre-existing ones, which in turn reduces the load-carrying capacity [16]. The significant reduction in strength can also be attributed to the gaps in the cement matrix created by the WAD substitution, which cause stress concentration and decrease the effective stress area [16]. Table 4 provides the variations in load and deformation. A display of the probability of crack width can be found in Figure 12. Furthermore, it can be noted that the mean value and standard deviation using Minitab [35] are calculated to be 7.08 and 7.17, respectively. Additionally, the scale effect is critical for the debonding mechanism of failure, and further testing on full-size beams is needed to validate the consistency of the present models at all relevant scale levels [36,37].

## 8. Analysis of FEM Processes

**Figure 13:**
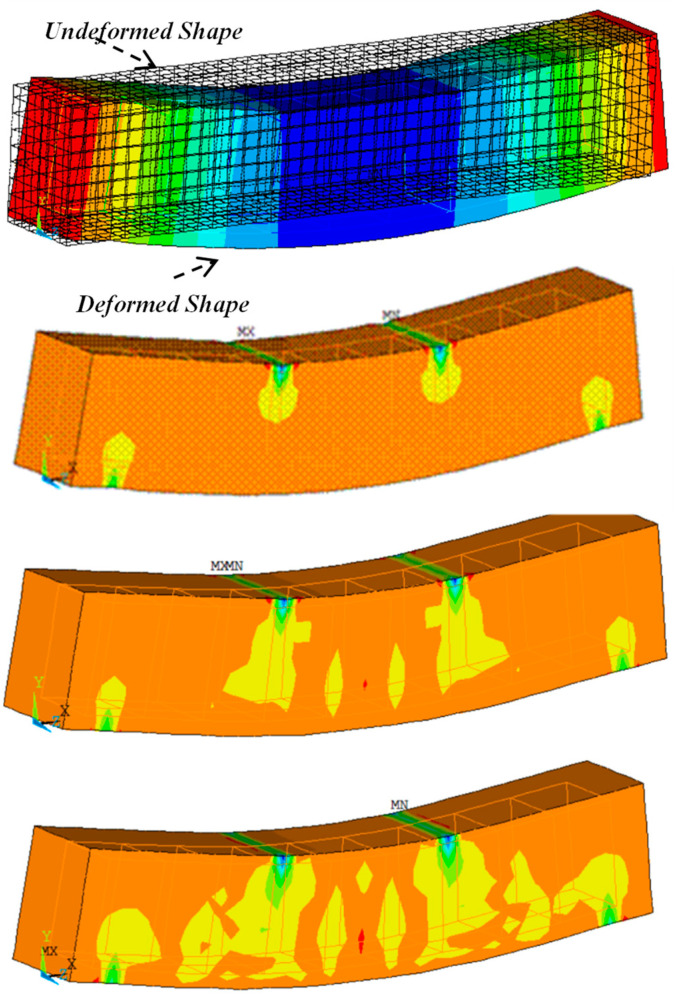
Bending behaviors of RCBs under perpendicular loads and stress attitudes of RCBs with 0% WAD.

This section presents the results of 3D FEM simulations conducted on several RCBs with varying proportions of WAD. To achieve this objective, a series of 3D evaluations were conducted on RCBs using five different proportions of WAD. The original RCB geometry, which was created using ANSYS based on FEM, is reflected in the 3D FEMs of the RCBs. Figure 13 displays the results of the 3D FEM for RCBs with 0% WAD. Figure 13 presents a comprehensive depiction of deformations and cracks specifically seen in the RCB. Stress distributions in the RCB under loads were obtained as shown in Figure 4. The RCB has significant flexural and vertical cracks.

**Figure 14:**
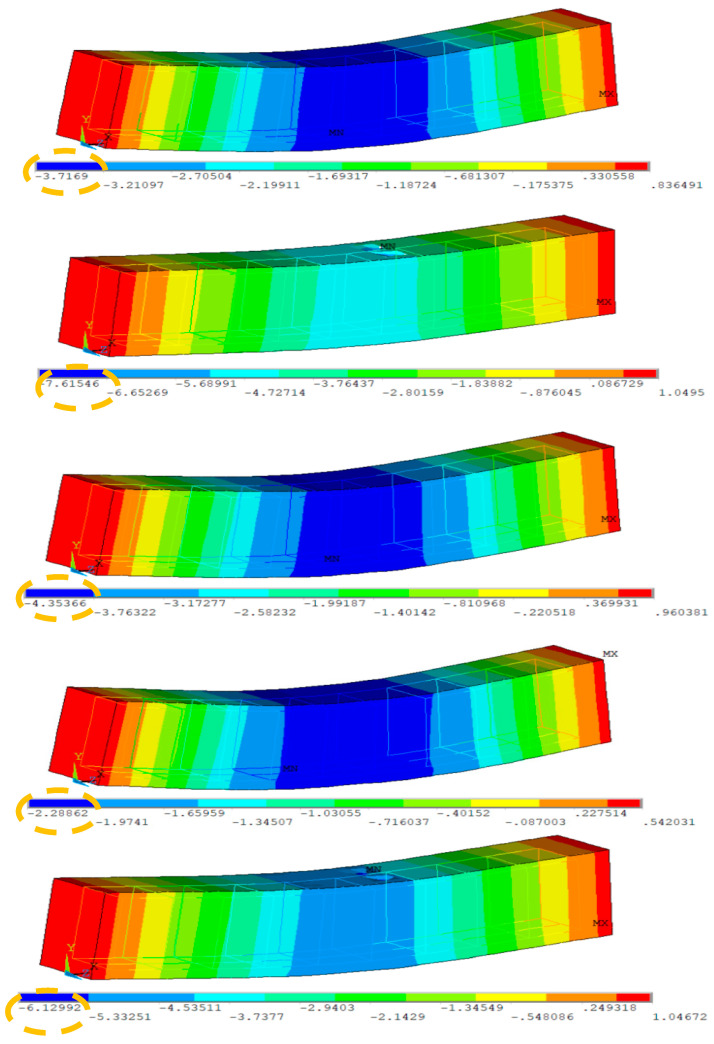
Experiments and simulations of vertical load deformation (cm).

Figure 14 provides the results of the RCBs for maximum deflection. The RCB’s flexing and deformation attitudes are displayed as contour plots in Figure 14. Noticeable disparities in fractures and deformations can be observed when comparing 3D FEM evaluations. As seen in previous FEMs, the midpoint of the RCB exhibited the highest estimated deviation in this particular model.

**Figure 15:**
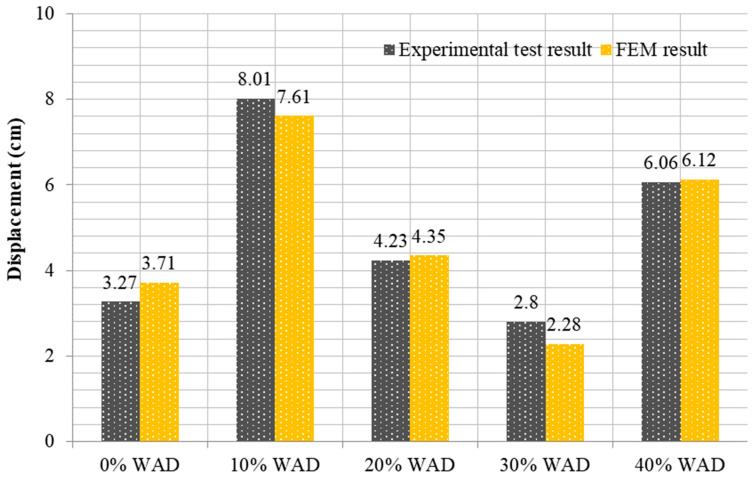
Comparative results of experimental and 3D numerical deformation data.

Comparing the findings obtained from the tests with the results obtained using the FEM is the focus of this section. As observed in Figure 14, upon examining the test results, it becomes evident that the maximum deformation for RCBs containing 0% WAD is measured as 3.27 cm. This value was obtained as 3.71 cm in the FEM. As a result, it is evident that 3D FEM results are established for the test outcomes of RCBs with 0% WAD. It appears that stress distributions in the RCB under loads are extremely similar to those discovered from the test outcomes, as shown in Figure 15. At the middle of the RCB with a 10% WAD rate, the maximum deformation is 8.01 cm. Based on the results of the test, a maximum deformation of 7.61 cm was observed, as shown in Figure 15. This outcome serves as clear evidence that the test results align with the predictions made using 3D FEM. It is evident that there is a clear recognition of the close proximity between the numerical and experimental fractures. With 20% WAD included, a 3D FEM was carried out for the RCBs. As seen in previous FEMs, the midpoint of the RCB exhibited the highest estimated deviation in this particular model. Conversely, the edge points of the RCBs containing 20% WAD had the lowest deviation. The maximum deflection of 4.23 cm was measured in the middle of the RCB. According to the results, a maximum deformation of 4.35 cm occurred in the middle of the RCB for 3D FEM analysis. Additional 3D FEM evaluations, including 30% and 40% WAD, were performed for the RCBs. For 30% and 40% WAD, respectively, the RCB deviation levels in FEM were 2.28 cm and 6.12 cm. The experimental testing revealed that the RCBs containing 30% and 40% WAD exhibited maximum deviations of 2.80 cm and 6.06 cm, respectively. Figure 15 presents a visual representation of both the empirical findings from the experimental tests and the 3D FEMs on the maximum displacement observed in the RCBs. In addition, Figure 15 presents the relationship between the results of the experiment and the FEM. The variability in material characteristics, such as the contact algorithm and yield strength on the contact surfaces, is assumed to be the primary explanation for the significant disparity between the experimental findings and the theoretical predictions.

## 9. Conclusions

This study examined the effects of various rates of WAD on RCBs. To achieve this objective, experimental and numerical analyses were conducted on the RCBs at varying rates of WAD, and the resulting deformations in the RCBs were monitored. As a result of the study, the following conclusions were obtained:

- The compression test results revealed that increasing WAD content in concrete consistently reduced its compressive strength. With no WAD, the compressive strength was 23.5 MPa, but it dropped to 22.2 MPa at 10% WAD and further declined to 16.8 MPa at 40% WAD. The reduction in compressive strength was particularly rapid beyond 20% WAD.
- A depletion in compressive strength can mainly be attributed to the dilution of cement because the efficiency factor drops as the andesite content increases. It is found that such an impact can hardly be compensated for by the small pozzolanic activity of andesite.
- The RCB containing 10% WAD exhibited slightly lower load-bearing and ductility capacities compared to reference beam; however, these properties experienced significant declines when the WAD content exceeded 10%. At 40% WAD, both load-bearing capacity and ductility were substantially reduced. Based on the experimental findings, replacing cement with 10% WAD is an optimum choice for producing eco-friendly concrete.
- With acceptable variations in the results, the results strongly suggest that FE modeling is an excellent alternative to laboratory experiments of RCBs with WAD. Therefore, it may be possible to accurately predict the behavior of RCBs in real-life scenarios using FE simulations.
- In light of the findings presented above, the recycling of WAD in mixes has the potential to have a beneficial influence on the elimination of the adverse environmental effects produced by waste storage areas created by natural-stone processing industries, as well as on the reduction of the costs associated with the manufacturing of materials.

### Recommendations for Future Work

Before considering WAD as a viable substitute for conventional aggregate-based concrete, further research and analysis are necessary. The chemical structures of the products in the WAD should be examined in future research using energy-dispersive X-ray photo spectroscopy (EDS) and scanning electron microscopy (SEM) analyses of the surface of the ruptured concrete, as well as the mechanism of strength change. Modifying some of the beams and columns inside the constructions may also enhance the earthquake-resistant performance of the building, extend the construction’s fundamental period, intensify the horizontal earthquake load, and deteriorate the lateral structural rigidity. Nevertheless, considering the fact that the use of WAD in a wide variety of materials is quite restricted, it is necessary to explore not only mechanical qualities but also other features. It is proposed that more research should be conducted to explore the impact of WAD on various concrete buildings or in a variety of environmental circumstances.

## Figures and Tables

**Table 1:** Different amounts of WAD.

Mix Number #	Statement
Mix 1	RCB including 0% WAD
Mix 2	RCB including 10% WAD
Mix 3	RCB including 20% WAD
Mix 4	RCB including 30% WAD
Mix 5	RCB including 40% WAD

**Table 2:** Mix proportion (1 m^3^).

Material (kg/m^3^)	WAD				
	0%	10%	20%	30%	40%
Cement	350	315	280	245	210
Sand	800	800	800	800	800
Coarse aggregate	920	920	920	920	920
Water	165	165	165	165	165
Andesite	0	80	160	240	320

**Table 3:** CEM I 42.5 R cement and andesite chemical and physical analysis [18].

Component	Cement	Andesite
SiO_2_ (%)	18.85	56.93
Al_2_O_3_ (%)	4.80	16.86
Fe_2_O_3_ (%)	2.40	5.39
CaO (%)	62.80	5.49
MgO (%)	2.50	2.89
Na_2_O + K_2_O (%)	1.14	9.89
SO_3_ (%)	3.69	—
Free CaO (%)	0.90	—
Loss on ignition	3.50	2.55
Density (g/cm^3^)	3.12	2.65

**Table 4:** The importance of conducting tests on load and displacement values.

Test No.	P_max_ (kN)	Def. at the P_max_ (mm)	*δ_u_* (mm)	Stiffness at P_max_ (kN/mm)	*P_u_* (0.85 *P_max_*) (kN)	Displ. at Yield *δ_y_* (mm)	Stiffness at Yield (kN/mm)	Ductility Ratio
Test #1	228.5	27.8	32.7	8.2	194.2	4.2	45.3	7.62
Test #2	193.9	28.8	80.1	6.7	164.8	11.9	13.8	6.70
Test #3	191.7	24.8	42.3	7.7	162.9	17.0	9.6	2.48
Test #4	184.3	11.9	28.0	15.4	156.6	9.5	16.5	2.96
Test #5	155.6	25.8	60.6	6.0	132.2	20.5	6.5	2.96

## Data Availability

Data will be made available on request.

## References

[1] Behforouz B., Memarzadeh P., Eftekhar M., Fathi F. (2020). Regression and ANN models for durability and mechanical characteristics of waste ceramic powder high performance sustainable concrete. Comput. Concr..

[2] Ansari M., Safiey A. (2020). Corrosion effects on mechanical behavior of steel fiber reinforced concrete, including fibers from recycled tires. Comput. Concr..

[3] Ren R., Qi L., Xue J., Zhang X., Ma H., Liu X., Ozbakkaloglu T. (2021). Concrete-steel bond-slip behavior of recycled concrete: Experimental investigation. Steel Compos. Struct. Int. J..

[4] Nematzadeh M., Baradaran-Nasiri A., Hosseini M. (2019). Effect of pozzolans on mechanical behavior of recycled refractory brick concrete in fire. Struct. Eng. Mech. Int’l J..

[5] Ali L., El Ouni M.H., Raza A., Kahla N.B. (2021). Investigation of FRP-reinforced recycled concrete compressive members: Experimental and theoretical analysis. Steel Compos. Struct. Int. J..

[6] Ozkilic Y.O., Zeybek O., ihsan Celik A., Althaqafi E., Mydin M.A.O., Dulaimi A., Karalar M., Jagadesh P. (2024). Fresh and hardened properties of expansive concrete utilizing waste aluminum lathe. Steel Compos. Struct..

[7] Almeshal I., Özkılıç Y.O., Aksoylu C., Karalar M., Alharthai M. (2024). Ductility and strength of reinforced concrete beams strengthened with aluminum CNC waste. Struct. Concr..

[8] Sariisik A., Sariisik G., Şentürk A. (2011). Applications of glaze and decor on dimensioned andesites used in construction sector. Constr. Build. Mater..

[9] Hamidi M., Kacimi L., Cyr M., Clastres P. (2013). Evaluation and improvement of pozzolanic activity of andesite for its use in eco-efficient cement. Constr. Build. Mater..

[10] Davraz M., Ceylan H., Topçu İ.B., Uygunoğlu T. (2018). Pozzolanic effect of andesite waste powder on mechanical properties of high strength concrete. Constr. Build. Mater..

[11] Labbaci Y., Abdelaziz Y., Mekkaoui A., Alouani A., Labbaci B. (2017). The use of the volcanic powders as supplementary cementitious materials for environmental-friendly durable concrete. Constr. Build. Mater..

[12] Kawabata Y., Dunant C., Yamada K., Scrivener K. (2019). Impact of temperature on expansive behavior of concrete with a highly reactive andesite due to the alkali–silica reaction. Cem. Concr. Res..

[13] Sogancioglu M., Yel E., Yilmaz-Keskin U.S. (2013). Utilization of andesite processing wastewater treatment sludge as admixture in concrete mix. Constr. Build. Mater..

[14] Çelikten S. (2021). Mechanical and microstructural properties of waste andesite dust-based geopolymer mortars. Adv. Powder Technol..

[15] Ceylan H., Davraz M. (2019). Atık andezit tozu ve uçucu küllerin betonda kullanımının karşılaştırılması. Doğal Afetler Çevre Derg..

[16] Özkan Ş., Ceylan H. (2022). The effects on mechanical properties of sustainable use of waste andesite dust as a partial substitution of cement in cementitious composites. J. Build. Eng..

[17] Soykan O., Özel C., Öcal C. (2015). Arduvaz ve Andezit’in Beton Agregası Olarak Kulanılabilirliğinin Araştırılması. Süleyman Demirel Üniversitesi Fen Bilim. Enstitüsü Derg..

[18] Eken M. (2023). Mechanical and Durability Properties of Concrete with Volcanic Rock Additives. J. Mater. Civ. Eng..

[19] Uzun İ. (2011). Andezitin Asfalt Betonunda Agrega Olarak Kullanılabilirliğinin Araştırılması.

[20] Uzun İ., Terzi S. (2012). Evaluation of andesite waste as mineral filler in asphaltic concrete mixture. Constr. Build. Mater..

[21] Orhan M., Işık N., Topal T., Özer M. (2006). Effect of weathering on the geomechanical properties of andesite, Ankara–Turkey. Environ. Geol..

[22] Özkan Ş. (2024). The impact of ternary hybrid fibers on the mechanical characteristics of cement-based composites with waste andesite dust substitution. J. Build. Eng..

[23] Bayraktar O.Y., Yakupoglu U., Benli A. (2023). Slag/diatomite-based alkali-activated lightweight composites containing waste andesite sand: Mechanical, insulating, microstructural and durability properties. Arch. Civ. Mech. Eng..

[24] Özkan Ş., Ceylan H. (2024). Atık Malzeme Olarak Uçucu Kül ve Andezit Tozu İçeren PVA Lif Donatılı Çimento Esaslı Kompozitlerin Basınç Dayanımının Tahmininde Yanıt Yüzey Metodolojisinin Kullanılması. Afyon Kocatepe Üniversitesi Fen Ve Mühendislik Bilim. Derg..

[25] Handayani A.F., Rosyidah D.H., Sulaksitaningrum R., Susanto P.B., Umniati S. (2023). Andesite waste powder as mineral admixture in concrete: A Review. E3S Web of Conferences.

[26] Çelikten S., Sarıdemir M., Soloğlu M. (2024). Effects of elevated temperatures and cooling regimes on the waste andesite dust-based geopolymer mortars. Constr. Build. Mater..

[27] Martín D.A., Costafreda J.L., Sanjuán M.A., Costafreda-Velázquez J.L. (2023). Mineral, Chemical and Technical Characterization of Altered Pyroxenic Andesites from Southeastern Spain for Use as Eco-Efficient Natural Materials. Appl. Sci..

[28] ANSYS R. (2020). Academic Research Mechanical, Release 19.2, Help System.

[29] Sabbağ N., Uyanık O. (2018). Determination of the reinforced concrete strength by apparent resistivity depending on the curing conditions. J. Appl. Geophys..

[30] Gemi L., Alsdudi M., Aksoylu C., Yazman S., Ozkilic Y.O., Arslan M.H. (2022). Optimum amount of CFRP for strengthening shear deficient reinforced concrete beams. Steel Compos. Struct. Int. J..

[31] Karalar M. (2020). Experimental and numerical investigation on flexural and crack failure of reinforced concrete beams with bottom ash and fly ash. Iran. J. Sci. Technol. Trans. Civ. Eng..

[32] Karalar M., Bilir T., Çavuşli M. 3D experimental and numerical investigation on crack behaviour of RC beams under% 75 bottom ash ratio. Proceedings of the Structures20 Congress.

[33] Karalar M., Ozturk H., Ozkilic Y.O. (2023). Experimental and numerical investigation on flexural response of reinforced rubberized concrete beams using waste tire rubber. Steel Compos. Struct..

[34] Korkut F., Karalar M. (2023). Investigational and numerical examination on bending response of reinforced rubberized concrete beams including plastic waste. Materials.

[35] Minitab Inc. (2014). MINITAB Release 17: Statistical Software for Windows.

[36] Casas J.R., Pascual J. (2007). Debonding of FRP in bending: Simplified model and experimental validation. Constr. Build. Mater..

[37] Ceci A.M., Casas J.R., Ghosn M. (2012). Statistical analysis of existing models for flexural strengthening of concrete bridge beams using FRP sheets. Constr. Build. Mater..

